# Supplementary material for: Trophic consequences of terrestrial eutrophication for a threatened ungulate
Source: Proc Biol Sci. 2021 Jan 20;288(1943):20202811. doi: 10.1098/rspb.2020.2811 (PMC7893279; doi:10.1098/rspb.2020.2811)
Supplement: Appendices for the main text [file rspb20202811supp1.pdf]

Electronic Supplementary Material for: Trophic consequences of terrestrial eutrophication for a threatened ungulate

Proceedings of the Royal Society of London B, Biological Sciences

DOI: 10.1098/rspb.2020.2811

Serrouya, R., M. Dickie, C. Lamb, H. van Oort, A. Kelly, C. DeMars, P.D. McLoughlin, N.C. Larter, D. Hervieux, A.T. Ford, S. Boutin.

APPENDIX S1. Pearson Correlation matrix among all variables for the 14 wolf survey units.

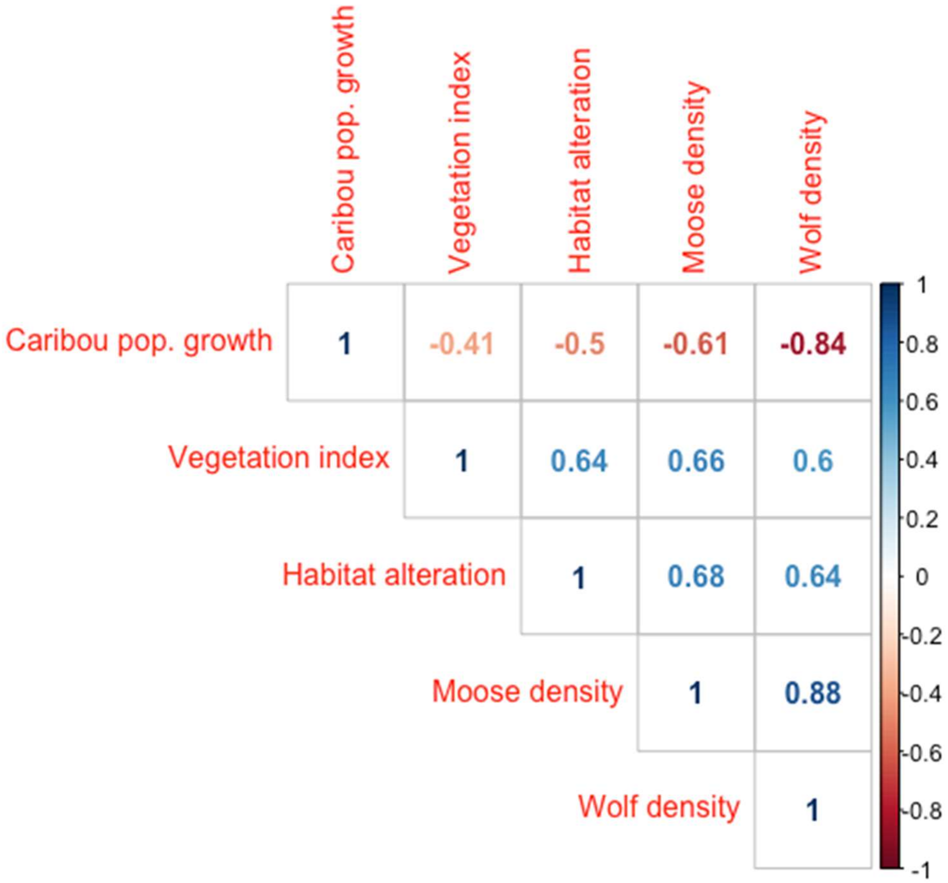

Figure S1-1. Pearson correlation matrix of all variables considered in the path analysis. Caribou pop.growth is the caribou population growth rate (λ), Habitat alteration is the % of wolf survey

unit that is altered by human activity, vegetation index is the ΔEVI (see main text for explanation). Code is presented at https://github.com/ctlamb/borealcaribou-pathanalysis

APPENDIX S2. Factors affecting the Enhanced Vegetation Index across the study area

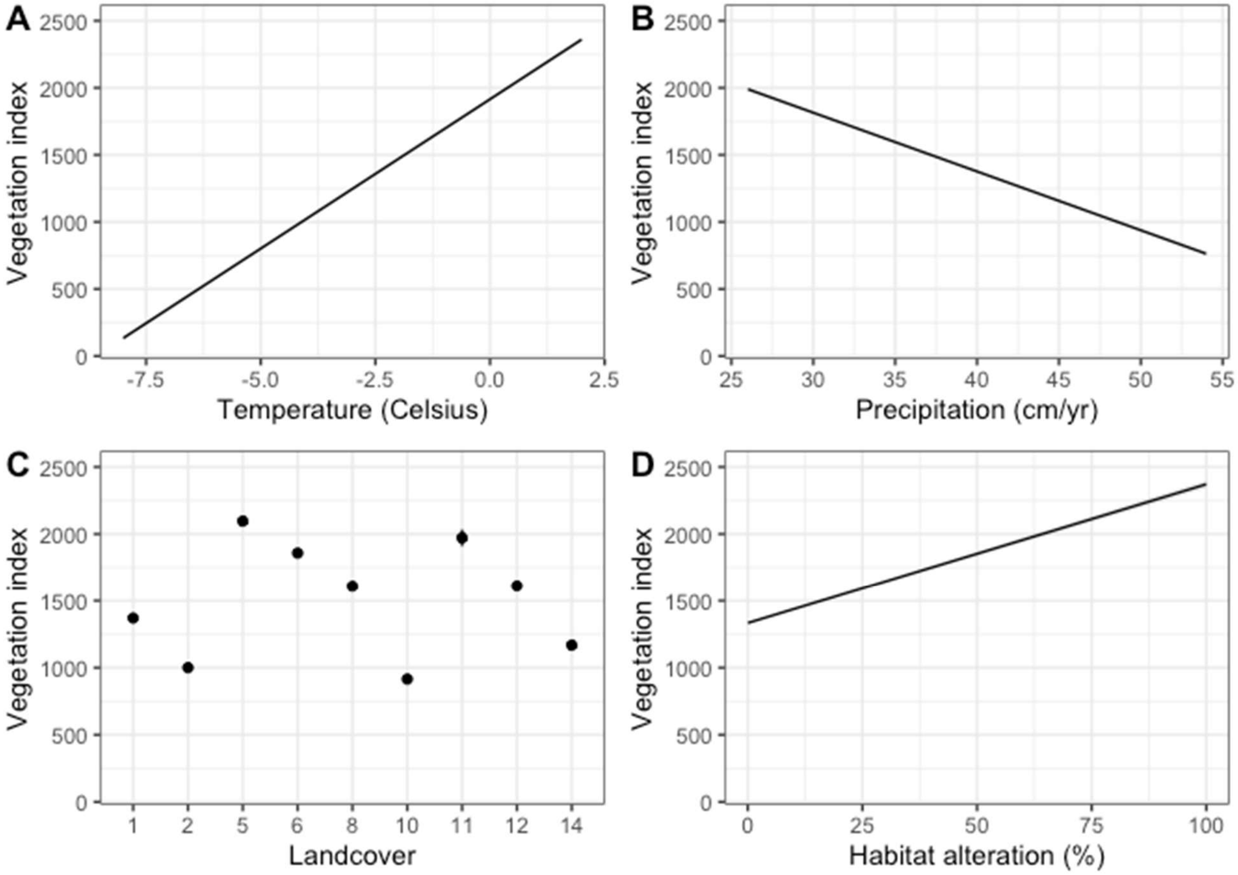

Figure S2-1: Factors predicting the Enhanced Vegetation Index (ΔEVI), including temperature (Celsius), precipitation (cm year^{-1}), landcover and habitat alteration (%). Predicted linear relationships are depicted for continuous variables, whereas landcover depicts the average ΔEVI for each landcover class. Landcover classes are: 1= Temperate or sub-polar needleleaf forest; 2= Sub-polar taiga needleleaf forest; 5= Temperate or sub-polar broadleaf deciduous forest; 6= Mixed forest; 8= Temperate or sub-polar shrubland;10= Temperate or sub-polar grassland; 11= Sub-polar or polar shrubland-lichen-moss;12= Sub-polar or polar grassland-lichen-moss; 14= Sub-polar or polar barren-lichen-moss.

We conducted a spatial analysis of factors that predict the vegetation index (ΔEVI) across the 598,000-km^2 study area. We used a linear model including all 500-m pixels in the study ($n > 127,000$). The R^2 was 0.44 ($F = 9293$, $df = 127088$, $p < 0.0001$). P-values are not meaningful with such high sample size, but the point was to show the magnitude of multiple factors affecting the vegetation index. This is why we did not link habitat alteration (on its own) directly to vegetation index in the path analysis (even though they are highly correlated, Appendix S1), because vegetation index interacts with landcover, temperature, and precipitation. Raw code and parameter estimates for this analysis are on GitHub: https://github.com/ctlamb/borealcaribou-pathanalysis/tree/master/seral_mechs_spatial

APPENDIX S3: Estimating Moose Densities

We obtained moose densities using aerial moose surveys conducted by provincial governments, academic, and industry partners between 2008 and 2018 (Table S3.1). Moose surveys were primarily conducted using either the ver Hoef (2008) geospatial or a stratified random block design (Gasaway 1986) but distance sampling became more frequently used as of 2010 (Buckland et al. 2004). Moose density estimates from aerial surveys were not available in the Cold Lake Saskatchewan Wolf Survey Unit (WSU). We therefore estimated the density of moose using remote wildlife cameras, and corrected camera estimates to aerial survey estimates using a correlation analysis. We first evaluated the relationship between moose densities estimated using remote wildlife cameras to densities estimated using aerial surveys across Alberta, and applied this correction factor to estimated moose densities in Saskatchewan from wildlife cameras.

Table S3.1: Estimated moose density (animals km^{-2}) in each Wolf Survey Unit (WSU). The year in which the estimate was calculated, method, and citation source are provided.

WSU #	WSU Name	Sub-Area	Moose Density	Year	Method	Citation
6	Fort Liard		0.0716	2017	Geospatial Population Survey	Larter, personal communication
8	Fort Providence South FMA		0.029	2012	Geospatial Population Survey	Kelly, personal communication
7	Fort Providence Reference		0.029	2012	Geospatial Population Survey	Kelly, personal communication
9	Fort Resolution FMA		0.013	2009	Geospatial Population Survey	Kelly and Cox, 2017
10	Fort Resolution Reference		0.013	2009	Geospatial Population Survey	Kelly and Cox, 2017
11	Hay River Lowlands		0.029	2012	Geospatial Population Survey	Kelly, personal communication
1	Calendar		0.018	2010	Distance Sampling	Theisen 2010
2	Chinchaga RRA		0.157	2016	Distance Sampling	Webster and Lavellee 2016

3	Clarke		0.074	2016	Distance Sampling	Webster and Lavellee 2016
5	Cold Lake Saskatchewan		0.0789	2017	cameras	unpublished data
12	Northern Saskatchewan		0.0457	2008-2015	Aerial surveys, various designs	McLoughlin et al, 2016
4	Cold Lake Alberta	WMU 529	0.089	2017	Distance Sampling	Government of Alberta, 2019
4		WMU 512	0.3	2013	Stratified Random block	Government of Alberta, 2018
4		WMU 519	0.14	2015	Distance Sampling	Government of Alberta, 2019
4		WMU 517	0.085	2018	Stratified Random block	Government of Alberta, 2019
13	Whati (TASR Impact)		0.011	2018	Distance Sampling	Kelly, personal communication
14	Jean Marie River		0.045	2018	Geospatial Population Survey	Kelly, personal communication

To compare density estimates for moose from cameras deployed, we related the estimated moose density from each of the provincial aerial surveys to estimated moose densities from a wildlife camera program deployed by the Alberta Biodiversity Monitoring Institute (ABMI) across Alberta’s boreal forest. The results can be used to correct camera density estimates for moose to the aerial survey estimates from government surveys within WSUs, to maintain consistency with density estimates used in the remainder of the analyses.

ABMI deployed cameras across 1197 sites from 2013 to 2018 across 38 Wildlife Management Units. Density estimates for moose were calculated for each ABMI camera, using the time-in-field-of-view method (Laurent et al. 2020), similar to that of methods presented in Nakashima *et al.* (2018). The time-in-field-of-view model uses cumulative time in the camera detection zone to estimate population density:

$$D = \frac{\sum(N \cdot T_F)}{A_F \cdot T_O}$$

Where density *D*, is calculated as the total number of individuals observed *N* multiplied by the time in front of the camera field-of-view T_F, divided by the area of the camera field-of-view A_F multiplied by the total camera operating time T_O. The units are animal-seconds per area-seconds, which equates to the number of animals per area.

The probability of detecting an animal decreases as the distance from the camera increases, and this is likely species- and habitat-specific. Therefore, the effective detection distance (*EDD*) in which each species, in each season was calculated using a prominently coloured pole 5 m from the camera. All animals were recorded as being closer or farther than the pole, with additional categories for animals that were uncertain (near 5 m but not directly in line with the pole), investigating the pole or investigating the camera. The effective detection distance was calculated using the proportion of locations that were < 5 m away versus > 5 m (excluding the uncertain and investigating images): EDD (m) = 5 / sqrt(1-$p_{>5m}$), where $p_{>5m}$ is the proportion of images with the species > 5 m away. The area surveyed by a camera is calculated as:

$$A_F = \frac{\pi \cdot EDD^2 \cdot \angle}{360}$$

Where A_F, in m^2, is calculated as π multiplied by *EDD*, multiplied by the camera field-of-view's angle in degrees, $\angle$, which is 42° with the cameras used here, all of which are divided by 360°.

Density estimates were calculated for summer and winter seasons and averaged with equal weight. Average moose density for each Wildlife Management Unit was calculated from all

cameras in the Wildlife Management Unit. Confidence intervals were calculated using a compound distribution of binomial presence/absence and log-normal abundance-given-presence. Aerial survey estimates were provided by the Government of Alberta. Estimates were provided with 90% confidence intervals.

We fit models of camera density as a function of aerial survey density, including Generalized Additive Models (GAMs) with smoothing splines using both normal and log-normal (log-link) error distributions, and a linear model both with and without an intercept. Points were weighted in inverse proportion to the width of the camera confidence intervals, which vary widely due to large differences in number of cameras per Wildlife Management Units and inherent variability of camera estimates. Confidence interval width for aerial estimates were a consistent proportion of the mean estimate, and so we did not weight aerial estimates.

We omitted one outlying datum with aerial density of 0.5 km^{-2} but camera density of 7.1 km^{-2}. The extreme camera estimate is from a Wildlife Management Units with only 4 cameras, and is largely due to a single camera with an extended visit from one moose. The 90% confidence intervals for that camera estimate are 1.4 – 35.3 km^{-2}, indicating an extremely uncertain estimate. We included one datum with an outlying aerial estimate of 0.77 km^{-2} in the analyses.

There was a general positive relationship between camera estimates and aerial-survey estimates of moose across Wildlife Management Units, but wide scatter as densities increase (Figure S3.1). The very wide confidence intervals on the GAM included the linear fit line. The linear models with and without intercepts were very similar. Because the models produced similar results and

the linear model without intercept is the simplest for developing a correction factor, we used the linear model. The correction factor, 1/slope of the linear model without intercept, was 0.478 (90% Confidence Interval: 0.415 – 0.568). The aerial estimate for moose density in a Wildlife Management Units is 0.478 times the camera estimate. Equivalently, the camera estimate is 2.09 times higher than the aerial estimate.

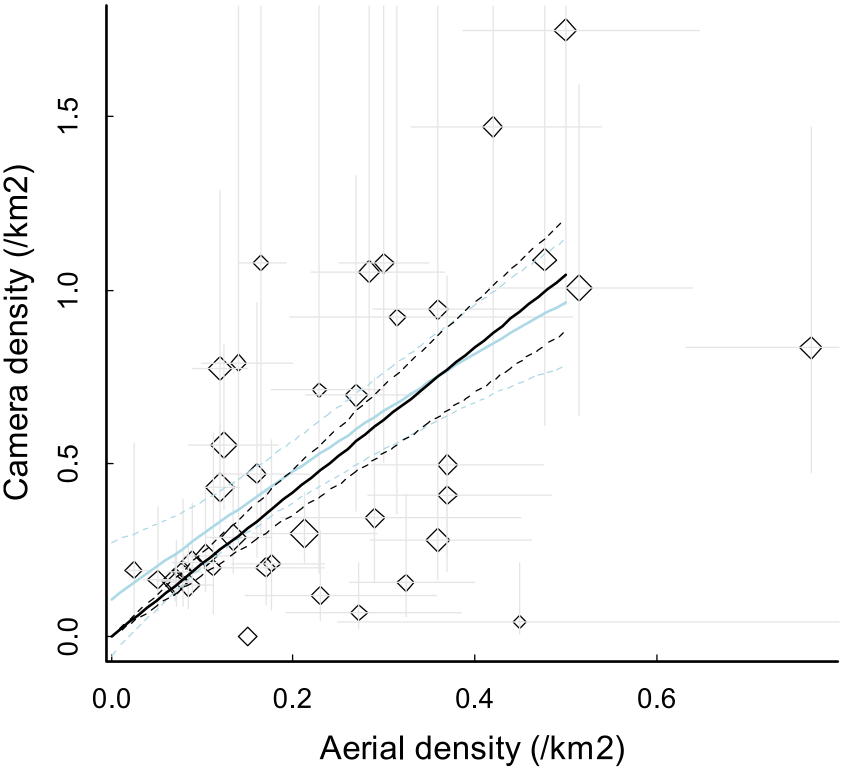

Figure S3-1: The relationship between moose density (moose km^{-2}) calculated using remote wildlife cameras and via aerial surveys across Alberta Wildlife Management Units. Thick black line represents a linear model with no intercept (dotted lines = 90% Confidence Intervals); pale blue line represents a normal GAM curve (pale grey dotted lines = 90% Confidence Intervals).

The substantial overestimation of moose densities by cameras is expected. ABMI cameras are put in open micro-habitats so that vegetation doesn't hide animals for at least 5 m; moose prefer those open areas for foraging, particularly in summer. Additionally, moose are attracted to the cameras themselves, often spending time investigating the camera. This inflates densities estimates by increasing the time that moose spend in the camera's field-of-view and also reduces the effective detection distance.

We applied the correction factor to moose densities estimated in Cold Lake Saskatchewan caribou range using remote cameras that overlapped the Cold Lake Saskatchewan WSU. Cameras in the Cold Lake Saskatchewan caribou range were randomly placed within a 12.5 x 4 -km area, with a minimum spacing of 1 km between each camera. Cameras collected data from January 2017 to March 2018. We calculated moose density using the approach as described above for each camera, and averaged across the 25 cameras to get one density estimate for that region. We estimated the moose density within the Cold Lake Saskatchewan WSU as 0.0789 moose km^{-2}. We then corrected the estimated density by multiplying by the correction factor, 0.478, such that 0.0789 moose km^{-2} * 0.478 = 0.0377 moose km^{-2} or 3.77 moose 100 km^{-2}.

References

Buckland ST, Anderson DR, Burnham KP, Laake JL, Borchers DL, Thomas L. 2004 *Advanced distance sampling*. Oxford University Press Oxford.

Gasaway WC. 1986 *Estimating moose population parameters from aerial surveys*. Fairbanks: Institute of Arctic Biology.

Laurent M, Dickie M, Becker, M, Serrouya R, & Boutin, S. 2020 Evaluating the Mechanisms of

Landscape Change on White-Tailed Deer Populations. *J. Wildlife Management*, 1–14. https://doi.org/10.1002/jwmg.21979

Nakashima Y, Fukasawa K, Samejima H. 2018 Estimating animal density without individual recognition using information derivable exclusively from camera traps. *J. Appl. Ecol*. 55, 735–744.

Ver Hoef JM. 2008 Spatial methods for plot-based sampling of wildlife populations. *Environ. Ecol. Stat.* **15**, 3–13.

APPENDIX S4: Estimating Wolf Densities: Spatial simulations to optimize transect spacing and time since snowfall for aerial surveys

We attempted to conduct a complete wolf census at each Wolf Survey Unit (WSU) based on the principle that independent wolf track networks (viewable track segments) will be isolated from each other and readily countable shortly after snowfall events. We conducted the survey by flying parallel transects, where the probability of intercepting track networks depended on transect spacing (survey intensity) and the size of the track network, which in turn was related to the time since snowfall. There is a trade-off between the expediency of a survey and the level of intensity at which the survey is conducted.

To inform survey intensity, and to understand how time elapsed since snowfall prior to surveying affected detection rates, we examined 12 wolf location time series, each from wolves collared with GPS collars, from different packs, with collars programmed to record a wolf location every 5 min. We considered only data from December through March to be consistent with winter survey conditions. Each time series included between 9 and 65 days of tracking (mean = 67 days per time series).

To simulate a survey, we extracted a segment from each time series to represent a network of observable tracks following a snowstorm. We chose the date of segment initiation and the number of days represented in each segment randomly. Track segments were 1, 2, or 3 days in length. We superimposed each track segment against a set of simulated survey transects that were always oriented north-south, positioned randomly in the east-west direction, and spaced 1,

2, 3, 5, or 7-km apart. Detection was determined if wolf track segments intercepted a survey transects at least once.

We repeated the simulated snow track segment outlined above 100 times for each time series. For each time series, we calculated the proportion of snow track segments that were detected for each combination of transect spacing and segment length. These proportions were presented using box plots. All programming was conducted in R using the following packages: rgdal, lubridate, plyr, reshape, and ggplot2.

As expected, detection rates increased when transect spacing was reduced and when the number of days included in a track segment increased (figures S4-1). The results indicated that 3 days of tracks are reliably intercepted using transect spacing from 1 to 3 km apart, and that 3-km spacing detects 91.6% of the track networks 2 days following a snowfall. We chose 3-km spacing based on this simulation.

These estimates are conservative because (1) this analysis was based on a single animal, whereas wolves travelling in packs have multiple tracks at times, and (2), old tracks are often evident under recent snowfall and these are also noted and considered when searching for fresh tracks. Finally, because WSUs were large and surveyed in one effort over several days, track detectability increased over time (e.g., 2, 3, 4, 5 etc. nights worth of tracks as survey progressed).

After wolf tracks were intercepted along a transect, the tracks were forward-tracked and sometimes back-tracked to count the number of wolves in the group using tracking evidence and

visual observations of the wolf packs. Each survey took approximately 3 to 5 days to complete, depending on weather and the size of the WSU.

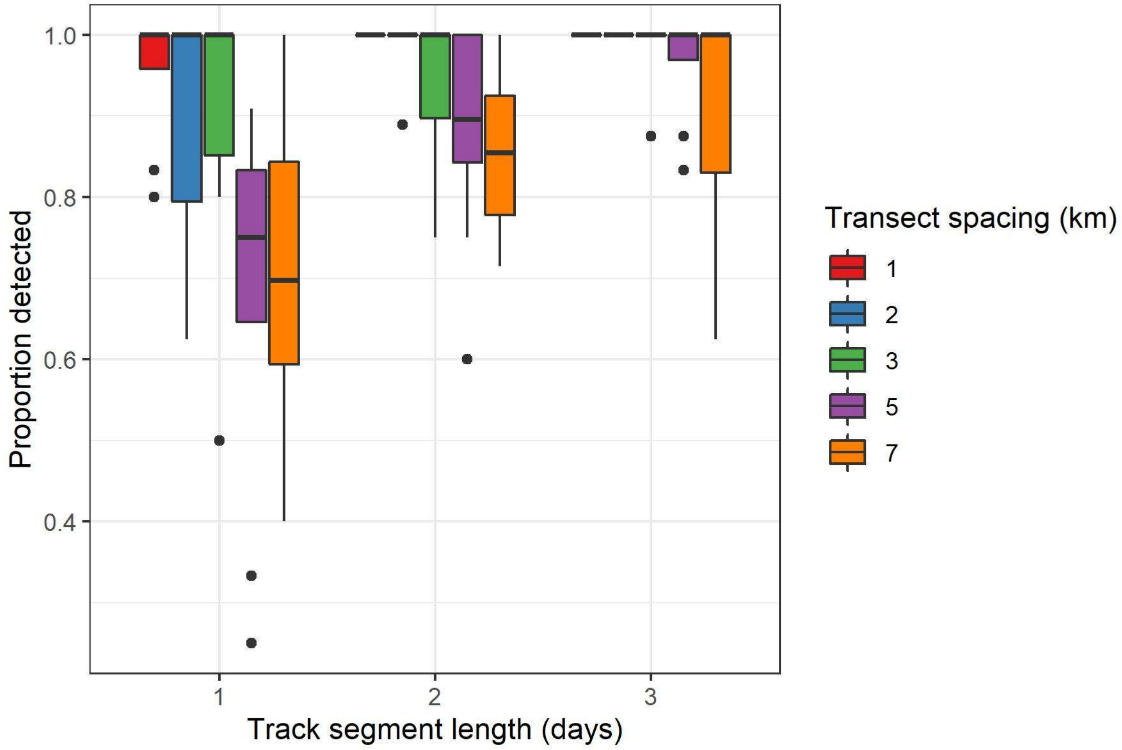

Figure S4-1. The proportion of track segments detected based on their length and transect spacing (km). The track segment length represents “time since snowfall” to guide when surveys should begin following a snowfall event.
